# Clinical and haemodynamic characteristics of preterm infants with early onset sepsis

**DOI:** 10.1111/jpc.16218

**Published:** 2022-09-17

**Authors:** Niall Johnston, Koert de Waal

**Affiliations:** ^1^ Department of Neonatology John Hunter Children's Hospital Newcastle New South Wales Australia

**Keywords:** haemodynamics, preterm infant, sepsis, septic shock

## Abstract

**Aim:**

Early onset sepsis (EOS) in preterm infants is associated with high morbidity and mortality. Improved characterisation of the haemodynamic presentation of EOS could lead to more effective and targeted treatments.

**Methods:**

We performed a retrospective observational study of preterm infants ≤32 week' gestation with EOS between 2010 and 2020 and gathered clinical, haemodynamic and echocardiographic data.

**Results:**

Of the 2198 admitted infants, 27 infants (median gestational age 28 weeks, median birthweight 1174 g) developed EOS with predominantly gram‐negative pathogens and the overall mortality rate was 33.3%. Besides hypotension, clinical signs were non‐specific, and over half of infants were normotensive in the first 72 h of life. Those with hypotension received more fluid resuscitation, inotropic support and had a higher mortality compared to the normotensive infants. Cardiac ultrasound was available in 18 infants and commonly revealed higher as expected cardiac output, pulmonary hypertension and diastolic dysfunction.

**Conclusion:**

Preterm infants with EOS had a high mortality rate, especially when they progressed from sepsis to septic shock. Echocardiography revealed a normal haemodynamic pattern, or one suggestive of vasodilatation and warm shock physiology. Targeting this pathophysiology earlier might improve outcomes.

## What is already known on this topic

1. Perinatal infection is a primary cause of neonatal death in Australia and world‐wide.
2. In particular, early onset sepsis (EOS) in preterm infants is associated with a high mortality.
3. Recognition of clinical sepsis in this group is challenging.

## What this paper adds

1. The clinical signs of preterm infants with EOS were difficult to distinguish from other causes of transitional hypotension.
2. Preterm infants with EOS did not present with a haemodynamic pattern of vasoconstriction and cold shock physiology, but blood flows were higher as expected for post‐natal age and suggestive of warm shock.
3. Targeting this pathophysiology with judicious fluids and vasoactive agents earlier might improve outcomes.
4. To identify septic shock early, a cardiac ultrasound in the first 6 h of life could be considered in preterm infants with risk factors for EOS, irrespective of clinical examination findings.

Early onset sepsis (EOS), or culture‐proven infection occurring at less than 72 h of age, is a major cause of morbidity and mortality in neonates. Studies have shown that those most at risk are very premature and/or very low‐birthweight infants. These high‐risk infants have an EOS incidence rate of 13.5 per 1000 live‐born very preterm infants,^1^ more than 20 times the incidence rate of term infants reported elsewhere.^2^ Despite extensive efforts to optimise care, EOS outcomes for premature infants remain associated with high mortality. EOS occurs when organisms that colonise the maternal genitourinary tract invade the fetal compartment through ruptured or intact membranes, causing intra‐amniotic infection or colonisation of the newborn around the time of birth. If bacterial infection becomes invasive, the infant will activate a pro‐inflammatory response, often with haemodynamic disturbances such as changes to the vascular barrier and progressive organ dysfunction.^3^, ^4^ Clinically, this could lead to either low cardiac output and high systemic vascular resistance which may manifest as cold skin, delayed capillary refill time and weak pulses (cold shock), or as high cardiac output and low systemic vascular resistance which may manifest with bounding pulses or flushed skin (warm shock). The time between introduction of the pathogen, the host immune response and the clinical presentation is highly variable in newborn infants. The cardiovascular transition from fetus to newborn is a complex haemodynamic situation where many changes in lung compliance, cardiac function and vascular tone are expected. There is limited data on the clinical and haemodynamic presentation of EOS in newborns, especially for preterm infants.^5^, ^6^ In studies of preterm neonates with late‐onset sepsis (LOS), the predominant haemodynamic presentation was high cardiac output and low systemic vascular resistance.^7^, ^8^ Our hypothesis was that preterm infants with EOS would behave similarly. Our aim in this retrospective observational study was to describe the clinical and haemodynamic characteristics of preterm infants with EOS.

## Methods

We performed a retrospective observational study in the neonatal intensive care unit of John Hunter Children's Hospital, Newcastle, New South Wales, Australia between 2010 and 2020. Our hospital is a tertiary‐care centre which has approximately 4000 deliveries per year. In addition to this, there are 11 rural birthing units as well as 6 large regional hospitals in the region that refer to unwell newborns. The study was approved by the local ethics committee.

Eligible infants included those born at or below a gestational age of 32 weeks with EOS, which was defined as a positive blood or cerebrospinal fluid bacterial culture within the first 72 h of life. Infants who developed sepsis outside this time window or those with significant congenital heart disease or other birth anomalies were excluded from the study.

Interpretation of cultures was as follows: a ‘positive’ culture was defined as single growth of pathogenic bacteria from blood or cerebrospinal fluid in a symptomatic infant, while a ‘contaminant’ culture was defined as single growth of coagulase‐negative staphylococcus, micrococci or other non‐pathogenic species with repeat negative culture within 48 h in an asymptomatic infant. Cultures demonstrating mixed growth in a symptomatic infant could be considered a ‘contaminant’ based on the opinion of the treating neonatologist as documented in the notes.

Electronic medical records from eligible infants were reviewed. Extracted data included maternal and birth records, clinical examination, microbiological results, interventions and outcomes. Due to the dynamic nature of EOS and the transition from fetus to newborn, some of the data were serially collected over seven time intervals or epochs within the first 72 h of life (0–6, 6–12, 12–18, 18–24, 24–36, 36–48 and 48–72 h).

Cardiac ultrasound performed in the first 72 h after birth were analysed to calculate blood flows (left ventricular output, right ventricular output, superior vena cava flow), left ventricular systolic function (Tissue Doppler s′ velocity, biplane method of disks ejection fraction) and left ventricular diastolic function (Doppler EA ratio, Tissue Doppler e′, the Ee′ ratio, left atrium volume). Persistent pulmonary hypertension of the newborn was explored by reviewing tricuspid valve regurgitant jet velocity, shunt direction over the ductus arteriosus and ventricular septal flattening.^9^ For the purpose of this study, we defined low and high blood flow as infants who showed values outside the reference range for post‐natal age.^10^, ^11^ Diastolic dysfunction was defined as an Ee′ ratio > 16 or Ee′ ratio > 14 with other indicators of diastolic dysfunction such as left atrium enlargement or EA ratio > 1.00.^12^

Hypotension was defined as a mean arterial pressure in mmHg less than gestational age in weeks for more than 15 min. For the purpose of this study, septic shock was defined as clinical poor perfusion, hypotension or cardiovascular dysfunction on ultrasound requiring treatment (fluids, vasopressors, inotropes, systemic steroids) in the presence of sepsis.

Local management of septic shock in our unit at the time advocated early fluid expansion (20 mL/kg bolus in 30 min, up to 60 mL/kg). In fluid‐refractory cases, a vasopressor agent such as noradrenaline or dopamine was recommended. Dosing was titrated based on the clinical picture provided by physical examination, blood pressure monitoring and cardiac ultrasound when available.

Descriptive data was gathered and analysed in Microsoft Excel. Data were graphically represented using GraphPad software (Version 8.4.3, Prism, San Diego, CA, USA) and one‐way ANOVA with multiple comparisons (mean of each column compared to all other columns) was used to explore changes over time and a *P* value < 0.05 was considered significant.

## Results

2198 infants born ≤32 weeks' gestation were admitted to the unit during the 10‐year study period. Thirty‐three blood cultures were positive within the first 72 h of life in this group, and 6 blood cultures grew coagulase‐negative *Staphylococcus* (1), *Micrococcus* species (3) and viridans streptococcal species (2) that were deemed contaminants. Twenty‐seven infants (12.2 per 1000 live preterm born) with positive blood cultures were included in the final analysis.

The antenatal and birth characteristics are presented in Table 1. Common risk factors for EOS in our group were prolonged preterm rupture of membranes (PPROM), positive or unknown group B streptococcus status and the presence of a cerclage. Infants with EOS were more commonly infected with gram‐negative pathogens (59.3%) compared to gram‐positive pathogens (40.7%). The most common gram‐negative species was *Escherichia coli* (12), followed by *Haemophilus influenzae* (4). The common gram‐positive pathogens were Group B streptococcus (3) and coagulase‐negative *Staphylococcus* (3). Other cultures included methicillin‐sensitive *Staphylococcus aureus* and *Streptococci* species.

**Table 1:** Clinical characteristics of preterm infants with early onset sepsis

Maternal factors	
PPROM	17 (63%)
GBS status positive or unknown	15 (56%)
Clinical and histological chorioamnionitis	9 (33%)
Cervical cerclage	5 (19%)
Antenatal and birth factors	
Complete antenatal steroid administration	17 (63%)
Male sex	11 (41%)
Aboriginal or Torres Strait Island heritage	6 (22%)
Gestational age (weeks)	28 (26–29)
Birthweight (g)	1174 (940–1400)
5‐min Apgar score less than 7	8 (30%)
Cord pH	7.27 (7.11–7.35)
Cord lactate (mmol/L)	3.0 (1.9–4.0)
Pathogens identified on blood culture	
Gram‐negative	16 (59%)
Gram‐positive	11 (41%)

Data presented as median (interquartile range) or *n* (%).

GBS, Group B streptococcus, PPROM, prolonged preterm rupture of membrane.

### Haemodynamic profile of preterm infants with EOS

**Fig. 1:**
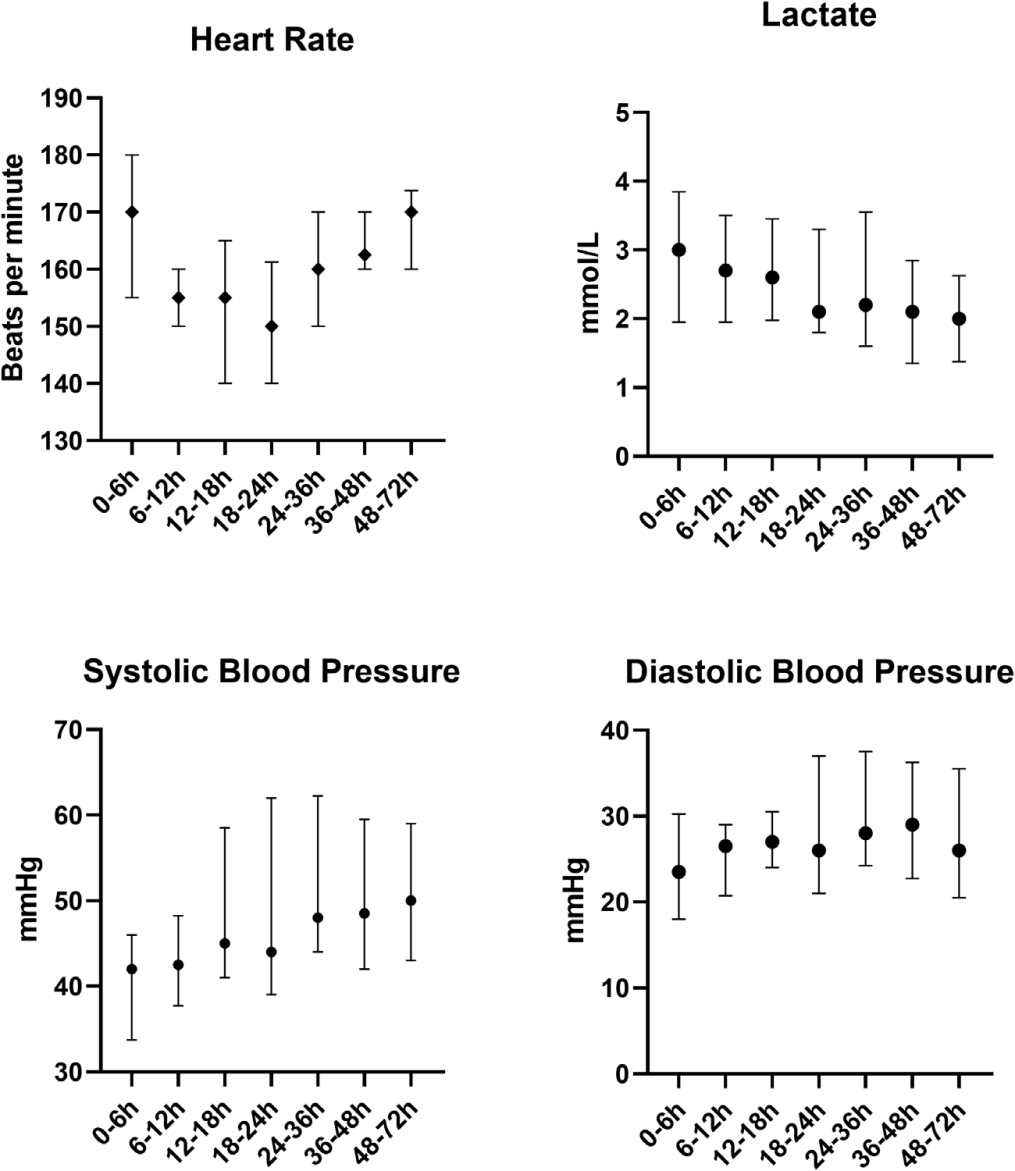
Heart rate, blood pressure and lactate in the first 72 h of life of 27 infants with EOS. Data presented as median and interquartile range. EOS, early onset sepsis.

The clinical signs in the first 6 h after birth were either non‐specific, or described respiratory deterioration and hypotension with or without poor perfusion. Figure 1 outlines haemodynamic parameters in the first 72 h of life. The median heart rate fluctuated between 150 and 170 bpm and changed significantly over the first 72 h of life (*F* = 5.75, *P* < 0.001). Significant tachycardia (>200 bpm) was seen in one infant in the first 6 h of life who received dopamine during this period. Median lactate values over the first 72 h of life ranged between 2.0 and 3.0 mmol/L (*F* = 0.67, *P* = 0.669) and the maximum lactate recorded was 19.0 mmol/L. The median ranges for systolic blood pressure, diastolic blood pressure and mean blood pressure were 42–51, 24–31 and 29–37 mmHg, respectively. Only systolic blood pressure changed significantly in the first 72 h after birth (*F* = 2.65, *P* = 0.018).

A slight majority of the infants with EOS (16 infants, 59%) were normotensive in the first 72 h of life. However, four were deemed to have clinical poor perfusion and received fluid treatment. Eleven infants (41%) developed sustained hypotension during the study period and received treatments. Ten of the 11 hypotensive infants received fluid boluses at a median (IRQ) volume of 30 (20–43) mL/kg and 7 infants received cardiovascular support with inotropes or vasopressors for septic shock, most starting within 24 h after birth. One infant received vasopressors for pulmonary haemorrhage on day 3.

### Echocardiography

Eighteen out of 27 infants (66.7%) had an echocardiogram performed at a median of 25 h (range 4–42) after birth. The infants who received a cardiac ultrasound tended to be more unwell. The cardiac ultrasound parameters are presented in Table 2. Ten (56%) infants presented with high blood flows and none had low central blood flows, 7 (39%) infants showed diastolic dysfunction, 5 with elevated Ee′ ratio > 16 and 2 with Ee′ ratio > 14 and an enlarged left atrium. Eight (44%) infants showed persistent pulmonary hypertension on ultrasound, all diagnosed as >30% right‐to‐left shunt over the ductus arteriosus. One infant had a low ejection fraction (42%) and all others had normal systolic function. Four infants received nitric oxide for hypoxia for pulmonary hypertension.

**Table 2:** Cardiac ultrasound findings in 18 preterm infants with early onset sepsis

Parameter (unit of measurement)	Median	Minimum	Maximum	Normal range
Right ventricular output (mL/kg/min)	299	166	675	170–320
Left ventricular output (mL/kg/min)	297	196	565	150–300
Superior vena cava flow (mL/kg/min)	108	66	156	50–120
Ejection fraction (%)	54	39	65	50–70
EA ratio	0.77	0.62	0.87	0.60–0.80
Ee′ ratio	16.5	12.5	18.8	7.0–14.0
Left atrium volume (mL/kg)	0.83	0.69	1.34	0.60–1.25

Data presented as median, minimum and maximum values.

### Mortality

The overall mortality rate in this cohort was 33.3%. Four infants died before 72 h and five died after 72 h. One normotensive infant died from pulmonary haemorrhage. Eight of the 12 hypotensive infants died, representing a mortality rate of 66.7% in this subgroup. An individual patient overview of clinical findings, cardiac ultrasound findings and outcomes are presented in Table 3.

**Table 3:** Clinical, cardiac ultrasound and outcome data for each patient

	Maximum FiO_2_	Mechanical ventilation	Maximum Lactate	Hypotension	Fluids in first 24 h	Vasoactive agents	Cardiac ultrasound	High blood flow	Diastolic dysfunction	Pulmonary hypertension	Outcome
**Patient 1**	30	No	2.0	No	No	No	No				Survived
**Patient 2**	21	No	3.7	No	No	No	No				Survived
**Patient 3**	40	Yes	2.0	No	No	No	Yes	No	No	No	Survived
**Patient 4**	30	No	11.3	No	Yes	No	Yes	Yes	Yes	No	Survived
**Patient 5**	21	No	1.5	No	No	No	Yes	No	No	No	Survived
**Patient 6**	21	No	3.9	No	No	No	No				Survived
**Patient 7**	30	No	2.3	No	No	No	No				Survived
**Patient 8**	30	Yes	3.5	No	No	No	Yes	No	No	No	Survived
**Patient 9**	21	No	2.7	No	No	No	Yes	No	No	No	Survived
**Patient 10**	55	No	2.9	No	No	No	No				Survived
**Patient 11**	40	No	3.6	No	No	No	Yes	No	No	No	Survived
**Patient 12**	23	No	2.4	No	No	No	Yes	No	No	No	Survived
**Patient 13**	40	No	5.1	No	No	No	Yes	No	No	No	Survived
**Patient 14**	40	No	8.7	No	Yes	No	No				Survived
**Patient 15**	60	Yes	4.6	No	No	No	No				Survived
**Patient 16**	70	No	2.8	Yes	No	No	No				Survived
**Patient 17**	57	Yes	7.6	Yes	Yes	Yes	Yes	Yes	No	Yes	Survived
**Patient 18**	90	Yes	3.8	Yes	No	No	No				Survived
**Patient 19**	40	Yes	3.0	Yes	No	Yes	Yes	Yes	No	Yes	Died
**Patient 20**	40	Yes	4.0	Yes	No	Yes	Yes	Yes	Yes	Yes	Died
**Patient 21**	50	Yes	6.8	Yes	Yes	No	Yes	Yes	Yes	Yes	Died
**Patient 22**	100	Yes	13.6	Yes	Yes	Yes	Yes	Yes	Yes	Yes	Died
**Patient 23**	100	Yes	4.9	Yes	Yes	Yes	Yes	Yes	Yes	Yes	Died
**Patient 24**	100	Yes	3.6	Yes	Yes	Yes	Yes	Yes	Yes	Yes	Died
**Patient 25**	40	Yes	2.9	Yes	Yes	Yes	Yes	Yes	Yes	No	Died
**Patient 26**	100	Yes	19.0	Yes	Yes	No	Yes	Yes	No	Yes	Died
**Patient 27**	35	Yes	1.1	No	No	Yes	Yes	No	No	No	Died

## Discussion

Various observational studies have assessed the haemodynamic response to LOS but few provided descriptive evidence of the haemodynamic presentation, clinical course of EOS and the progression to septic shock in this vulnerable age group.^5^, ^6^, ^7^, ^8^, ^9^, ^10^, ^13^, ^14^, ^15^ Our clinical and ultrasound observational data revealed that preterm infants with EOS presented with either normal clinical findings and haemodynamic cardiac ultrasound parameters within the reference range, or with hypotension and higher as expected blood flows suggestive of warm shock. Contrary to what was found in newborn animal studies of EOS, no infant in our study showed clinical or ultrasound evidence of cold shock.^16^, ^17^ Besides hypotension and increased respiratory support requirements, no specific clinical signs were predictive of progression of sepsis towards septic shock and mortality. We hypothesise that the difference in clinical presentation reflects the variation in exposure duration to pathogens *in‐utero* and the individual host response, whereby the initial host response is vasodilatation and warm shock physiology. Only when this cannot be maintained cold shock will follow, similar to sepsis pathophysiology in adults.^18^

Recognition of clinical sepsis is one of the main pillars to help reduce mortality in sepsis.^19^ However, clinical signs associated with sepsis are difficult to distinguish from the transitional changes after preterm birth. Combined with the fact that limited baseline data will be available, that is, there are only a few hours of clinical data for the clinicians to determine changes in blood pressure, heart rate or urine output, the clinical diagnosis of EOS in very preterm infants remains a challenge. Early start of antibiotics remains recommended after very preterm birth with risk factors for sepsis.

In our cohort, 59% of preterm infants with EOS progressed from sepsis to septic shock and this was associated with a significant increase in mortality. Considering the high mortality and the difficulty in diagnosing EOS clinically, one could argue that all preterm infants with risk factors for EOS should undergo routine assessment of vasodilatory changes, either with cardiac ultrasound or with alternative methods such as near‐red infrared spectroscopy.^7^, ^8^, ^20^ A major advantage of an early cardiac ultrasound would be to identify signs of vasodilatory shock as soon as it develops. Early detection would facilitate important, timely interventions such as insertion of arterial lines, central venous access, judicious fluid management and early use of vasopressors.

The approach of early diagnosis of sepsis phenotypes and earlier targeted interventions has led to improved outcomes in adults with septic shock.^21^ Adults with septic shock were categorised into hypovolemia and increased cardiac function, left or bi‐ventricular dysfunction and isolated right ventricular dysfunction, and treatments were directed to these three underlying pathophysiology phenotypes. Fluid therapy in all types was guided by ultrasound to prevent common fluid overload that occurs with sepsis resuscitation, and observational in children and randomised data in adults showed that early start of noradrenaline in sepsis can prevent severe prolonged hypotension, optimise cardiac output, recover the microcirculation, prevent fluid overload and improve clinical outcomes including mortality.^22^, ^23^, ^24^ This data aligns with a recent artificial intelligence study in which an advanced deep learning trained computer algorithm recommended that patients with sepsis should have been given vasopressors at an earlier time point to reduce sepsis mortality.^25^ Clearly more data is needed in the neonatal population. Ultrasound‐guided fluid management is mentioned in the latest adult and paediatric sepsis guidelines, but strategies to achieve this goal are still in the developmental phase for newborns.^26^, ^27^ Starting noradrenaline in a neonatal setting also comes with several additional invasive treatments like placement of a central line and need for an arterial line, but in a disease where mortality reaches 66%, this novel clinical approach warrants attention.

Our data showed that 1/3 of infants with EOS did not receive a cardiac ultrasound. Training and accreditation of bedside clinicians using this monitoring technique have increased in the last decade, but it remains difficult to provide around‐the‐clock cover in the neonatal setting. Given the limited time window and frequent haemodynamic changes in septic shock, there is a pressing need for more on‐site staff with bedside cardiac ultrasound skills. One proposed model of care might be to use on‐call neonatal experienced cardiac ultrasonographers. This model of care will likely lead to more general acceptance of integrating cardiac ultrasound findings into sepsis management. Developing a local cardiac ultrasound service will allow a collection of real time, longitudinal point of care information, assisting the clinician to understand the cardiac function and hemodynamic changes. This in turn allows targeting of cardiovascular support and monitoring of the response to treatment.

There are several limitations to this study. This retrospective observational study gathered clinical and haemodynamic data from the medical records. Despite our high standard of medical documentation in our unit, we acknowledge the possibility of incomplete data impacting results. Secondly, few serial echocardiograms were performed and not in all patients which could also impact conclusions. Despite this, our data suggest that very preterm infants with EOS present with either warm shock or no symptoms of shock at all. Careful review with serial cardiac ultrasounds can help reveal early signs of septic shock, and earlier treatment needs further evaluation to help reduce the high mortality of EOS in very preterm infants.

## Conclusion

Preterm infants with EOS have a high mortality, expecially when progressing from sepsis to septic shock. Echocardiography can help phenotype sepsis and guide treatments.
